# *FOXO3* polymorphisms influence the risk and prognosis of rhabdomyosarcoma in children

**DOI:** 10.3389/fonc.2024.1387735

**Published:** 2024-04-24

**Authors:** Xiaohong Zhang, Yaping Sun, Huilin Niu, Ping Tan, Shanshan Liu, Xiaoping Liu, Xiaodan Liu, Ailing Luo, Mansi Cai, Yaping Yan, Ling Xu, Xu Yang

**Affiliations:** ^1^ Department of Hematology/Oncology, Guangzhou Women and Children’s Medical Center, Guangzhou Medical University, Guangzhou, China; ^2^ Research Institute of Tsinghua University in Shenzhen, Shenzhen, China; ^3^ Department of Pathology, Guangzhou Women and Children’s Medical Center, Guangzhou Medical University, Guangzhou, China; ^4^ School of Pharmaceutical Sciences, Jilin University, Changchun, China; ^5^ Guangzhou Institute of Pediatrics, Guangzhou Women and Children’s Medical Center, Guangzhou Medical University, Guangdong Provincial Clinical Research Center for Child Health, Guangzhou, China

**Keywords:** FOXO3, single nucleotide polymorphisms (SNPs), rhabdomyosarcoma (RMS), susceptibility, prognosis

## Abstract

**Background:**

Rhabdomyosarcoma(RMS) is the most common soft tissue sarcoma in children and single nucleotide polymorphisms(SNPs) in certain genes influence risk of RMS. Although *FOXO3* had been reported in multiple cancers including RMS, the role of *FOXO3* polymorphisms in RMS remains unclear. In this case-control study, we evaluated the association of *FOXO3* SNPs with RMS risk and prognosis in children.

**Methods:**

Four *FOXO3* SNPs(rs17069665 A>G, rs4946936 T>C, rs4945816 C>T and rs9400241 C>A) were genotyped in 110 RMS cases and 359 controls. The associations between *FOXO3* polymorphisms and RMS risk were determined by odds ratios(ORs) with 95% confidence intervals(CIs). The associations of rs17069665 and rs4946936 with overall survival in RMS children were estimated using the Kaplan-Meier method and log-rank test. Functional analysis in silico was performed to estimate the probability that rs17069665 and rs4946936 might influence the regulation of *FOXO3*.

**Results:**

We found that rs17069665 (GG vs. AA+AG, adjusted OR=2.96; 95%CI [1.10-3.32]; *P*=0.010) and rs4946936 (TC+CC vs. TT, adjusted OR=0.48; 95%CI [0.25-0.90]; *P*=0.023) were related to the increased and decreased RMS risk, respectively. Besides, rs17069665(*P*<0.001) and rs4946936(*P*<0.001) were associated with decreased and increased overall survival in RMS patients, respectively. Functional analysis showed that rs17069665 and rs4946936 might influence the transcription and expression of *FOXO3* via altering the bindings to MYC, CTCF, and/or RELA.

**Conclusions:**

This study revealed that *FOXO3* polymorphisms influence the RMS susceptibility and prognosis in children, and might altered the expression of *FOXO3*. *FOXO3* polymorphism was suggested as a biomarker for RMS susceptibility and prognosis.

## Introduction

Rhabdomyosarcoma (RMS) is a myogenic tumor that arises from cells committed to skeletal muscle differentiation (1, 2). Although relatively rare, RMS is the most common soft tissue sarcoma and the third most prevalent extracranial solid tumor in children (3, 4). Despite of developments in the therapy and management of RMS patients in the past decades, the etiology of RMS remains unclear and the prognosis of RMS is also still poor (3, 5). Cancer occurs due to environmental exposure and/or genetic factors. In RMS initiation and development, genetic change has been suggested to play a vital role (3). One prevalent type of genetic variation in human disease is single nucleotide polymorphisms (SNPs). SNPs of certain genes have been validated to influence the incidence and outcome of multiple cancers including RMS (6, 7). However, many SNPs in genes associated with RMS are still not discovered and need to be explored.

Forkhead Box O3(*FOXO3*), also called Forkhead in Rhabdomyosarcoma-Like 1 (FKHRL1), belongs to the forkhead box O (FoxO) family of transcription factors which regulate the transcription of genes involved in multiple cellular processes (proliferation, apoptosis, autophagy, *etc.*) *(* 8–10). Evidence supported that the aberrant expression or dysfunction of FOXO3 was associated with various types of cancers including RMS (11–14). In addition, gene polymorphism of *FOXO3* has been validated to influence various human disorders including cancer, such as pancreatic cancer, colorectal cancer, active tuberculosis, polycystic ovary syndrome, and acute lymphoblastic leukemia (ALL) (15–20). However, the association of FOXO3 polymorphisms with childhood RMS risk and outcome has never been reported.

In this case-control study, we conducted genotyping assay for FOXO3 SNPs (rs17069665, rs4946936, rs4945816, and rs9400241) and estimated the association of FOXO3 polymorphisms with RMS risk and prognosis in children. Besides, the underlying mechanisms involved in FOXO3 regulation by these polymorphisms were explored via functional analysis in silico.

## Materials and methods

### Study subjects

In this current study, a total of 110 RMS cases and 359 non-cancer healthy controls were recruited from Guangzhou Women and Children’s Medical Center (GWCMC). Briefly, patients under 18 years old with RMS were identified with histological confirmation and enrolled in this study by pediatric clinicians. Each case was newly diagnosed and provided a detailed medical record. Patients with other malignancies, secondary disorders, or chemotherapy/radiotherapy records were excluded. Thus, 110 children with RMS were recruited during June 2012 to June 2019, and 359 age- and sex-matched healthy volunteers were collected as controls. The controls were randomly selected from children receiving routine physical examinations. All individuals included in this study were ethnic Han Chinese. Before participation, a written informed consent for each case was obtained in advance. This study was conducted under the guideline of the Institutional Review Board of GWCMC.

### SNP Selection and genotyping

In this study, four SNPs were selected using data from SNPinfo and NCBI dbSNP databases as described previously (21–23). Briefly, the selection was based on three criteria as the following: (1) SNPs with the minor allele frequency (MAF) >5% in Chinese population; (2) located in or near the FOXO3 gene (i.e., 3’-UTR or 5’-UTR); (3) affecting splicing activity, transcription factor binding sites (TFBS) or miRNA-binding sites activity. At last, rs17069665 A>G, rs4946936 T>C, rs4945816 C>T, and rs9400241 C>A were retrieved for further analyses.

For genotyping, DNA was extracted from peripheral blood sample of each participant using the TIANamp DNAKit (TianGen, Beijing, China). In the genotyping assays, the corresponding SNP probes and primers (ABI, Massachusetts, USA) were used to genotype these four SNPs as previously described (21). For quality control, 10% samples were selected randomly for direct sequencing (24, 25), and a concordance rate of 100% was obtained.

### Functional analysis in silico

The associations of rs17069665 A>G and rs4946936 T>C with FOXO3 expression were determined using the expression quantitative trait loci (eQTL) analysis in GTEx portal (https://www.gtexportal.org/home/) (26). The probability that these two polymorphisms might influence the regulation of FOXO3 was estimated by using the Roadmap Epigenome Browser (27, 28), the ENCODE Project (29, 30), and TFBIND software (31). In brief, promoter and enhancer were predicted via DNase hypersensitivity (DHS) and histone modification of muscle cells in Roadmap Epigenomics data. TFBIND was used to evaluate whether FOXO3 polymorphism rs17069665 A>G or rs4946936 T>C altered any TFBS, and then ENCODE ChIP-seq experiments of MYC, CTCF, and RELA (Experiment Series: ENCSR000DLR, ENCSR000ANS, ENCSR000EBD) were used to assess the binding signals and motifs overlapping rs17069665 or rs4946936.

### Statistical analyses

The demographic variables and genotype distribution of each SNP between the RMS case and control groups were compared using a 2-sided χ 2 test. For each SNP in the control group, Hardy-Weinberg equilibrium (HWE) was assessed using goodness-of-fit chi-square test. The strength of the association between FOXO3 polymorphism and RMS susceptibility was estimated using logistic regression analysis with odds ratios (ORs) and 95% confidence intervals (CIs), adjusting for age and sex. False-positive report probability (FPRP) was conducted for significant findings, as described previously (32). We adopted OR of 2.00 and 0.50 for risk and protective effects, respectively. FPRP values < 0.2 were considered noteworthy. Overall survival was estimated with Kaplan-Meier method, and then differences between subgroups were compared using the log-rank test. All statistical analyses were performed with SAS software and GraphPad Prism software; a two-sided *P* < 0.05 was considered to be statistically significant.

## Results

### Characteristics of subject

Totally, 110 RMS cases and 359 healthy controls were finally enrolled in this study. The detailed demographic characteristics are all summarized in **Table 1**. Briefly, no significant difference in the age (*P* = 0.947) or sex (*P* = 0.613) distribution was found between cases and controls. According to histological classification, 74 (67.27%) cases were diagnosed as embryonal RMS (ERMS), 27 (24.55%) cases as alveolar RMS (ARMS), and 9 (8.18%) cases as other subtypes including anaplastic and mixed RMS. In addition, other information including risk level, clinical stage, and site of origin were also included in **Table 1**.

**Table 1:** Frequency distribution of selected characteristics in RMS cases and cancer-free controls.

Variables	RMS Cases (n=110)			Controls (n=359)		*P* ^a^
	No.	%		No.	%	
Age range, years	0.10-13.9			0.50-13.0		0.947
Mean ± SD	3.21 ± 3.10			4.85 ± 2.38		
<10	104	94.55		340	94.71	
≥10	6	5.45		19	5.29	
Sex						0.613
Female	44	40.00		134	37.33	
Male	66	60.00		225	62.67	
Histological subtype						
Embryonal	74	67.27				
Alveolar	27	24.55				
Others	9	8.18				
Risk level						
Low	14	12.73				
Medium	44	40.00				
High	52	47.27				
Site						
Head and neck	11	10.00				
Trunk and limbs	33	30.00				
Genitourinary system	19	17.27				
Thoracic, abdominal and pelvic cavities	42	38.18				
Others	5	4.55				
Clinical stage						
I	7	6.36				
II	14	12.73				
III	63	57.27				
IV	21	19.09				
NA	5	4.55				

SD, standard deviation; NA, not available.

a Two-sided χ^2^ test for distributions between RMS cases and cancer-free controls.

### Associations between FOXO3 gene polymorphisms and RMS susceptibility

According to the SNP selection strategy, four SNPs (rs17069665 A>G, rs4946936 T>C, rs4945816 C>T, and rs9400241 C>A) that overlap with TFBS or miRNA-binding sites were selected (**Supplementary Table S1**). The genotype frequencies of FOXO3 SNPs in all 110 cases and 359 controls and their association with RMS risk were displayed in **Table 2**. All these four SNPs were in HWE (*P*_HWE_ > 0.05) among the control. Significant differences between RMS cases and controls were observed for rs17069665 A>G (*P* = 0.011) and rs4946936 T>C polymorphism (*P* = 0.025) in a recessive (GG vs. AA+AG) and a dominant (TC + CC vs. TT) model, respectively. After adjustment with age and sex, all these 2 polymorphisms, namely rs17069665 G allele (GG vs. AA+AG: adjusted OR = 2.96; 95% CI [1.10-3.32]; *P* = 0.010) and rs4946936 C allele (TC+CC vs. TT: adjusted OR=0.48; 95%CI [0.25-0.90]; *P*=0.023), were significantly related to the increased and decreased RMS risk, respectively. The remaining 2 polymorphisms (rs4945816 C>T and rs9400241 C>A), however, were not found to be in association with RMS risk.

**Table 2:** Logistic regression analysis of associations between *FOXO3* polymorphisms and RMS susceptibility.

Genotype	Cases (N=110)	Controls (N=359)	*P* ^a^	Crude OR (95% CI)	*P*	Adjusted OR (95% CI) ^b^	*P* ^b^
rs17069665 (HWE=0.7104, A/G: wt/mut)							
AA	71 (64.55)	241 (67.13)		1.00		1.00	
AG	28 (25.45)	105 (29.25)		0.91 (0.55-1.48)	0.693	0.90 (0.55-1.47)	0.668
GG	11 (10.00)	13 (3.62)		**2.87 (1.23-6.69)**	**0.014**	**2.89 (1.24-6.73)**	**0.014**
Additive(GG vs. AG vs. AA)			0.158	1.29 (0.91-1.83)	0.159	1.31 (0.92-1.87)	0.165
Dominant(AG+GG vs. AA)	39 (35.45)	118 (32.87)	0.615	1.12 (0.72-1.76)	0.615	1.12 (0.71-1.75)	0.633
Recessive(GG vs. AA+AG)	99 (90.00)	346 (96.38)	**0.008**	**2.96 (1.29-6.80)**	**0.011**	**2.98 (1.29-6.86)**	**0.010**
rs4946936 (HWE=0.3811, T/C: wt/mut)							
TT	17 (15.45)	29 (8.08)		1.00		1.00	
TC	37 (33.64)	158 (44.01)		**0.40 (0.20-0.80)**	**0.010**	**0.39 (0.19-0.78)**	**0.010**
CC	56 (50.91)	172(47.91)		0.55 (0.28-1.09)	0.086	0.55 (0.28-1.08)	0.083
Additive (CC vs. TC vs. TT)			0.542	0.91 (0.66-1.25)	0.542	0.91 (0.66-1.25)	0.560
Dominant (TC + CC vs. TT)	93 (84.55)	330 (91.92)	**0.023**	**0.48 (0.25-0.91)**	**0.025**	**0.48 (0.25-0.90)**	**0.023**
Recessive (CC vs. TT + TC)	54 (49.09)	187 (52.09)	0.582	1.13 (0.74-1.73)	0.582	1.14 (0.74-1.76)	0.542
rs4945816 (HWE=0.4398, C/T: wt/mut)							
CC	15 (13.64)	30 (8.36)		1.00		1.00	
CT	38 (34.55)	158 (44.01)		0.49 (0.24-1.15)	0.145	0.49 (0.24-1.13)	0.139
TT	57 (51.82)	171(47.63)		0.67 (0.34-1.33)	0.248	0.67 (0.34-1.33)	0.248
Additive (TT vs. CT vs. CC)			0.878	0.98 (0.70-1.35)	0.878	0.98 (0.71-1.36)	0.902
Dominant (CT + TT vs. CC)	95 (86.36)	329 (91.64)	0.442	1.18 (0.77-1.81)	0.442	1.19 (0.78-1.84)	0.410
Recessive (TT vs. CC + CT)	53 (48.18)	188 (52.37)	0.100	0.58 (0.30-1.12)	0.103	0.57 (0.30-1.11)	0.099
rs9400241 (HWE=0.7986, C/A: wt/mut)							
CC	16 (14.55)	30 (8.36)		1.00		1.00	
CA	38 (34.54)	151 (42.06)		0.47 (0.26-1.27)	0.347	0.46 (0.23-1.27)	0.324
AA	56 (50.91)	178 (49.58)		0.59 (0.30-1.16)	0.127	1.70 (0.86-3.36)	0.124
Additive (AA vs. CA vs. CC)			0.500	0.89 (0.65-1.23)	0.500	0.89 (0.65-1.24)	0.513
Dominant (CA +AA vs. CC)	94 (85.45)	329 (91.64)	0.056	0.54 (0.28-1.03)	0.059	0.53 (0.28-1.02)	0.056
Recessive (AA vs. CC+ CA)	54 (49.09)	181 (50.42)	0.808	1.06 (0.69-1.61)	0.808	1.07 (0.69-1.63)	0.774

a χ^2^ test for genotype distributions between RMS cases and cancer-free controls.

b Adjusted for age and gender.

wt/mut, wild-type/mutation.

The bold values were statistically significant results.

### Subgroup and stratification analyses

To further explore the association of the FOXO3 gene rs17069665 A>G and rs4946936 T>C polymorphisms with RMS susceptibility, we performed subgroup and stratification analyses in terms of age, sex, histological classification, risk level, clinical stage, and site of origin. As shown in **Table 3**, rs17069665 (GG vs. AA+AG) and rs4946936 (TC+CC vs. TT) were both associated with RMS in male (adjusted OR=3.33, 95% CI [1.16-9. 57], *P* =0.026; adjusted OR=0.36; 95% CI [0.17-0.78], *P*=0.009; respectively), in children aged < 10 years (adjusted OR=2.98, 95% CI [1.29-6.87], *P* =0.011; adjusted OR=0.45; 95% CI [0.24-0.86], *P*=0.016; respectively), in children with tumor of stage II (adjusted OR=9.95, 95% CI [2.73-36.23], *P* =0.001; adjusted OR=0.16; 95% CI [0.05-0.50], *P*=0.002; respectively), and in children with tumor in genitourinary system (adjusted OR=5.03, 95% CI [1.29-19.57], *P* =0.020; adjusted OR=0.25; 95% CI [0.08-0.73], *P*=0.012; respectively). In addition, rs17069665 (GG vs. AA+AG) was related to RMS in children with ERMS (adjusted OR=3.59, 95% CI [1.47-8.76], *P* =0.005), in children with tumor in high risk level (adjusted OR=3.57, 95% CI [1.28-9.90], *P* =0.015), in children with tumor of stage IV (adjusted OR=4.65, 95% CI [1.19-18.22], *P* =0.028), and in children with tumor in trunk and limbs (adjusted OR=4.00, 95% CI [1.20-13.34], *P* =0.024). Rs4946936 (TC+CC vs. TT) was related to RMS in children with tumor in medium risk level (adjusted OR=0.33, 95% CI [0.14-0.76], *P* =0.009) and in children with tumor in head and neck (adjusted OR=0.24, 95% CI [0.06-0.95], *P* =0.043). In the FPRP analysis (**Supplementary Table S2**), most of the above significant findings remained noteworthy at the prior probability level of 0.25(FPRP<0.200), which further strengthen the significant associations of rs17069665 and rs4946936 with RMS.

**Table 3:** Subgroup and stratification analysis of *FOXO3* polymorphisms with RMS susceptibility.

Variables	rs17069665 (cases/controls)		Adjusted OR ^a^	*P* ^a^	rs4946936 (cases/controls)			Adjusted OR ^a^	*P* ^a^
	AA+AG	GG	(95% CI)		TT	TC+CC		(95% CI)	
Age, years									
<10	93/327	11/13	**2.98 (1.29-6.87)**	**0.011**	17/28		87/312	**0.45 (0.24-0.86)**	**0.016**
≥10	6/19	0/0	0.36 (0.05-2.50)	–	0/1		6/18	999 (0.01-999)	0.984
Gender									
Female	40/129	4/5	2.48 (0.64-9.72)	0.191	4/10		40/124	0.81 (0.24-2.72)	0.729
Male	59/217	7/8	**3.33 (1.16-9. 57)**	**0.026**	13/19		53/206	**0.36 (0.17-0.78)**	**0.009**
Histological subtype									
Embryonal	65/346	9/13	**3.59 (1.47-8.76)**	**0.005**	11/29		63/330	0.51 (0.24-1.07)	0.075
Alveolar	26/346	1/13	1.11 (0.14-8.87)	0.925	5/29		22/330	0.36 (0.12-1.03)	0.056
Others	8/346	1/13	3.61(0.41-31.76)	0.247	1/29		8/330	0.69 (0.08-5.75)	0.730
Risk level									
Low	12/346	2/13	4.59(0.92-22.90)	0.063	2/29		12/330	0.52 (0.11-2.45)	0.410
Medium	41/346	3/13	1.83 (0.50-6.77)	0.365	9/29		35/330	**0.33 (0.14-0.76)**	**0.009**
High	46/346	6/13	**3.57 (1.28-9.90)**	**0.015**	6/29		46/330	0.68 (0.27-1.73)	0.415
Site									
Head and neck	10/346	1/13	2.52(0.30-21.15)	0.396	3/29		8/330	**0.24(0.06-0.95)**	**0.043**
Trunk and limbs	29/346	4/13	**4.00(1.20-13.34)**	**0.024**	3/29		30/330	0.83 (0.24-2.91)	0.767
Genitourinary system	16/346	3/13	**5.03(1.29-19.57)**	**0.020**	5/29		14/330	**0.25(0.08-0.73)**	**0.012**
Thoracic, abdominal and pelvic cavities	39/346	3/13	2.00 (0.54-7.34)	0.297	6/29		36/330	0.54 (0.21-1.39)	0.203
Others	5/346	0/13	0.01 (0.01-999)	0.985	0/29		5/330	999 (0.01-999)	0.977
Clinical stage									
I	7/346	0/13	0.01 (0.01-999)	0.981	2/29		5/330	0.20(0.04-1.14)	0.069
II	10/346	4/13	**9.95(2.73-36.23)**	**0.001**	5/29		9/330	**0.16(0.05-0.50)**	**0.002**
III	59/346	4/13	1.78 (0.56-5.66)	0.331	5/29		58/330	1.00 (0.37-2.69)	0.996
IV	18/346	3/13	**4.65(1.19-18.22)**	**0.028**	4/29		17/330	0.37 (0.12-1.20)	0.097

a Adjusted for age and sex.

The bold values were statistically significant results.

### Overall survival analyses

**Figure 1:**
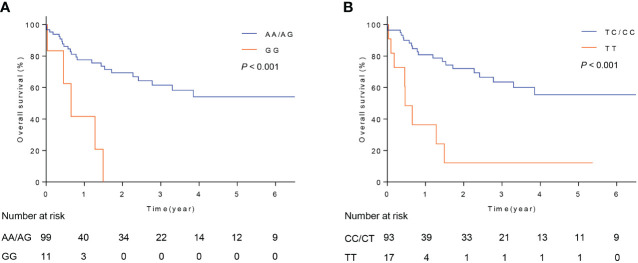
Overall survival of patients with RMS. **(A)** Survival curve of RMS patients with rs17069665 allele AA/AG or GG; **(B)** Survival curve of RMS patients with rs4946936 allele TC/CC or TT.

The above results indicated that FOXO3 polymorphisms, rs17069665 and rs4946936, influenced RMS risk in children. However, it’s not clear whether they influence the prognosis of RMS. Therefore, we evaluated the associations of rs17069665 and rs4946936 with overall survival in RMS patients. The results were described in **Figure 1**. For rs17069665, RMS patients with GG allele had a poor overall survival than those with AA/AG (**Figure 1A**, *P* < 0.001). Compared with rs4946936 TT, TC/CC allele significantly contributed to the increased overall survival in RMS patients (**Figure 1B**, *P* < 0.001). The results suggested that FOXO3 polymorphisms were associated with the prognosis in RMS children.

### Functional analysis

**Figure 2:**
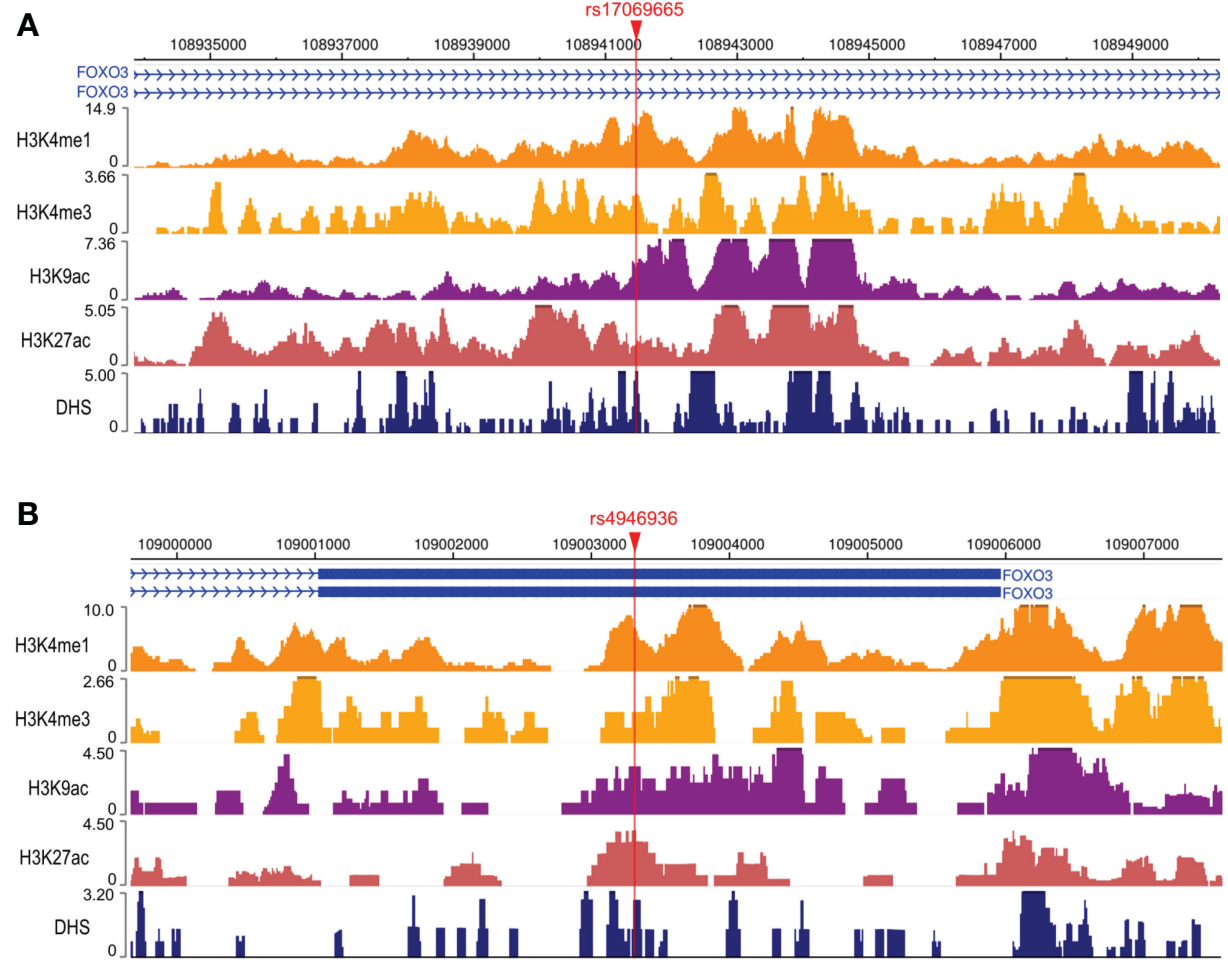
Rs17069665 and rs4946936 both overlap promoter and enhancer of FOXO3 gene. H3K4me1, H3K4me3, H3K9ac, H3K27ac, and DHS ChIP-seq signals at rs17069665 **(A)** and rs4946936 **(B)** loci.

**Figure 3:**
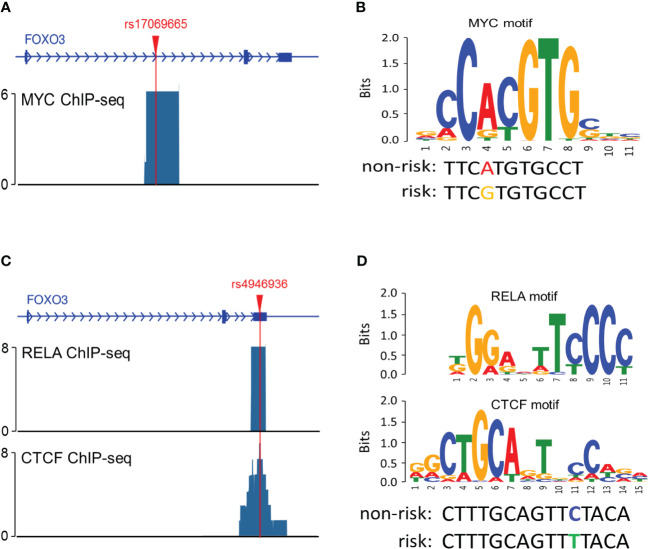
Rs17069665 and rs4946936 modulated the binding to MYC, RELA and/or CTCF. **(A)** MYC ChIP-seq signal at the rs17069665 locus; **(B)** Predicted preferential binding of MYC to the non-risk allele A of rs17069665; **(C)** RELA and CTCF ChIP-seq signals at the rs4946936 locus; **(D)** Predicted preferential bindings of RELA and CTCF to the non-risk allele C of rs4946936.

To explore the possible mechanisms by which rs17069665 and rs4946936 influence the risk and prognosis of RMS, we evaluated the probability of rs17069665 and rs4946936 polymorphism altering expression regulation of FOXO3. In the eQTL analysis, rs17069665 G allele was associated with lower expression of FOXO3 gene (*P* = 8.52×10^-4^) (**Supplementary Figure S1A**). Rs4946936 C allele displayed a trend of high expression of FOXO3, although no significance was achieved (*P* = 3.32×10^-1^) (**Supplementary Figure S1B**). The Roadmap Epigenomics data showed that rs17069665 (**Supplementary Figure S2A**) and rs4946936 (**Supplementary Figure S2B**) both overlapped DHS marks and histone modifications related to the enhancer and promoter in multiple tissue types. These were further supported by H3K9ac, H3K27ac, H3K4me1, H3K4me3, and DHS ChIP data in muscle cells (**Figures 2A, B**). TFBIND analysis showed that rs17069665 altered the binding affinity to transcription factors including ARNT, BHLHE40, MYC, MYOD, NKX2-5 and USF. Rs4946936 altered the binding affinity to CAP1, CTCF, MYB, RELA and SETDB1. However, ENCODE ChIP-seq analysis showed that only MYC binds to DNA motif overlapping rs17069665 (**Figure 3A**). MYC has a higher preference for the non-risk allele A than risk allele G (**Figure 3B**). As for rs4946936, only CTCF and RELA bind to DNA motif overlapping it (**Figure 3C**). CTCF and RELA have higher preferences for the non-risk allele C than risk allele T (**Figure 3D**). The results revealed that rs17069665 and rs4946936 might influence the transcription of FOXO3 via altering the bindings to MYC, CTCF and/or RELA.

## Discussion

In this case-control study with 110 RMS cases and 359 healthy controls, we evaluated the potential association of FOXO3 gene polymorphisms with RMS risk and prognosis in Chinese children. Among these four SNPs of FOXO3 in this study, we found that rs17069665 (GG vs. AA+AG) was significantly associated with increased RMS risk and poor prognosis, rs4946936 (TC+CC vs. TT) was significantly associated with decreased RMS risk and good prognosis. To our knowledge, the current study is the first one exploring the association between FOXO3 polymorphisms and RMS.

As a member of FOXO transcription factor family, FOXO3, as well as FOXO1, -4 and -6, controls transcription of target genes by binding to the Forkhead Response Element (33–35). Compared with the other three FOXO members, FOXO3 seemed to be more predominant in controlling cancer progression, although they potentially regulate the same target genes (36, 37). FOXO3 has been demonstrated to regulate various genes playing key roles in multiple cellular process including proliferation, apoptosis, drug resistance and stem cell properties (38–42). For instance, FOXO3 impaired the cancer stem cell phenotype of squamous cell carcinoma by controlling the transcriptional activity of SOX2 (11). The activation of FOXO3 sensitized tumors to anti-PD-1 therapy by inhibiting c-Myc and STAT3 (43). FOXO3 inhibited cell proliferation and induced apoptosis in colorectal cancer by regulating BIM expression (44). Certainly, FOXO3 is also regulated at multiple levels including genetic regulation and epigenetic modification (transcription, post-transcription, translation and post-translation) (40). FOXO3 protein was phosphorylated by proteins including ERK and AKT, which resulted in its inactivation and degradation (37, 45). FOXO3 gene polymorphisms were also investigated in multiple cancers, such as leukemia, pancreatic cancer, and hepatocellular carcinoma (16, 17, 46). However, FOXO3 polymorphisms have never been reported in RMS. It is urgent to conduct relevant investigations.

In the present study, we explored the association of four FOXO3 SNP sites (rs17069665, rs4946936, rs4945816 and rs9400241) with RMS. Rs17069665 was previously reported to increase ALL susceptibility (17), but had not been reported in other cancers. Rs4946936 was found to be associated with ALL (47), thyroid cancer (48), and head and neck cancer (49), but not with hepatocellular carcinoma (46). In this study, we found that rs17069665 and rs4946936 polymorphisms were associated with the increased and decreased RMS risk, respectively, and influence prognosis, for the first time. Rs17069665 is located in the intron one of FOXO3 gene, which overlaps with the FOXO3 promoter and enhancer. In the analyses of transcription factor binding, rs17069665 was found to disrupt the binding to MYC showing preferential binding of the non-risk allele A. Rs4946936, which is located in the 3’-UTR of FOXO3, was also found to overlap with the promoter and enhancer regions and to alter the binding to CTCF and RELA showing preferential binding of the non-risk allele C. SNP-gene expression analysis indicated that these two polymorphisms influenced the expression of FOXO3. Considering the tumor suppressing function of FOXO3, rs17069665 and rs4946936 might influence RMS risk and prognosis via regulating the expression of FOXO3 by altering the bindings to MYC, CTCF and/or RELA. The above potential mechanisms need to be validated in future studies.

As for the rest two SNPs (rs4945816 and rs9400241), both of which are located in the 3’UTR of FOXO3, no association between them and RMS risk was found. Several previous studies investigated the relationships between them and cancer risks. Rs4945816 was reported in studies on ALL and thyroid cancer, but no association was found (17, 48). Rs9400241 was associated with ALL and melanoma risks (17, 50). Along with the previous studies, our study indicates that the genetic variation of FOXO3 is complex, depending on cancer types. Besides, the variety in ethnicity and sample composition need to be taken into consideration.

Although this study is the first to investigate the association of FOXO3 polymorphisms with RMS risk and prognosis, several limitations should be considered. First, all participates were recruited from one hospital in south China, which may cause selection bias. Second, only 4 SNPs were genotyped in the present study and more potentially functional SNPs should be done in the future. Third, the sample size in this study was still not large enough because of the low incidence of RMS in China. Therefore, larger multicenter studies are warranted to further confirm the roles of FOXO3 in RMS. Finally, other factors including environment exposure and dietary intake were not available in this study. The functions of FOXO3 polymorphisms in the progression of RMS also need to be further explored.

## Conclusion

In conclusion, the current study explored the association of FOXO3 polymorphisms (rs17069665, rs4946936, rs4945816 and rs9400241) with RMS in Chinese children and firstly demonstrated that rs17069665 was associated with increased RMS susceptibility and poor prognosis, while rs4946936 was associated with decreased RMS susceptibility and good prognosis, and that the two polymorphisms might influence the transcription of FOXO3 via altering the binding to MYC, CTCF and/or RELA. This study indicated that FOXO3 polymorphism might serve as a biomarker for RMS susceptibility and prognosis. Certainly, larger multicenter studies, as well as functional experiments, are encouraged to further elucidate the role of FOXO3 polymorphism and the underlying mechanisms in RMS.

## Data availability statement

The original contributions presented in the study are included in the article/**Supplementary Material**. Further inquiries can be directed to the corresponding authors.

## Ethics statement

The studies involving humans were approved by Institutional Review Boards of Guangzhou Women and Children’s Medical Center. The studies were conducted in accordance with the local legislation and institutional requirements. Written informed consent for participation in this study was provided by the participants’ legal guardians/next of kin.

## Author contributions

XZ: Conceptualization, Writing – original draft, Writing – review & editing, Methodology, Data curation, Formal analysis, Validation. YS: Writing – original draft, Writing – review & editing, Conceptualization, Data curation, Formal analysis, Methodology, Funding acquisition. HN: Writing – original draft, Writing – review & editing, Investigation. PT: Writing – original draft, Writing – review & editing, Investigation. SL: Investigation, Writing – original draft, Writing – review & editing. XPL: Investigation, Writing – original draft, Writing – review & editing. XDL: Investigation, Writing – original draft, Writing – review & editing. AL: Investigation, Writing – original draft, Writing – review & editing. MC: Investigation, Writing – original draft, Writing – review & editing. YY: Investigation, Writing – original draft, Writing – review & editing. LX: Writing – original draft, Writing – review & editing, Conceptualization, Funding acquisition, Supervision. XY: Conceptualization, Funding acquisition, Supervision, Writing – original draft, Writing – review & editing, Methodology, Project administration.

## Supplementary material

Supplementary Figure 1 Functional relevance of rs3738067 A>G **(A)** and rs4946936 T > C **(B)** to FOXO3 expression in GTEx database.

Supplementary Figure 2 Schematic of rs17069665 **(A)** and rs4946936 **(B)** regions with histone and DHS mark annotations in different tissue types in the Roadmap epigenomics data.
