# Supplementary material for: FOXO3 polymorphisms influence the risk and prognosis of rhabdomyosarcoma in children
Source: Front Oncol. 2024 Apr 24;14:1387735. doi: 10.3389/fonc.2024.1387735 (PMC11076676; doi:10.3389/fonc.2024.1387735)
Supplement: Supplementary file 1 [file Table_1.docx]

| **Supplementary Table S1 SNPs captured by the four selected FOXO3 polymorphisms as predicted by dbSNP and SNPinfo** | | | | | | | |
| --- | --- | --- | --- | --- | --- | --- | --- |
| rs | Chr | Allele | TFBS / miRNA | Gene | Allele | Asian | CHB |
| rs17069665 | 6 | A/G | Y | FOXO3 | A | 0.92 | 0.93 |
| rs4945816 | 6 | C/T | Y | FOXO3 | T | 0.729 | -- |
| rs4946936 | 6 | C/T | Y | FOXO3 | T | 0.202 | 0.173 |
| rs9400241 | 6 | A/C | Y | FOXO3 | C | 0.222 | -- |
