# Supplementary material for: FOXO3 polymorphisms influence the risk and prognosis of rhabdomyosarcoma in children
Source: Front Oncol. 2024 Apr 24;14:1387735. doi: 10.3389/fonc.2024.1387735 (PMC11076676; doi:10.3389/fonc.2024.1387735)
Supplement: Supplementary file 2 [file DataSheet_1.docx]

| **Supplemental Table S2**. **False-positive report probability analysis for the significant findings** | | | | | | | | |
| --- | --- | --- | --- | --- | --- | --- | --- | --- |
| Genotype | Crude OR (95% CI) | *P* ^a^ | Statistical power^b^ | Prior probability | | | | |
|  |  |  |  | 0.25 | 0.1 | 0.01 | 0.001 | 0.0001 |
| rs17069665 A > G | | | | | | | | |
| GG vs. AA/AG | 2.96 (1.29-6.80) | 0.011 | 0.178 | **0.151** | 0.348 | 0.855 | 0.983 | 0.998 |
| Age <10 years | 2.98 (1.29-6.87) | 0.010 | 0.175 | **0.151** | 0.349 | 0.855 | 0.983 | 0.998 |
| Male | 3.33 (1.16-9. 57) | 0.026 | 0.172 | 0.308 | 0.572 | 0.936 | 0.993 | 0.999 |
| Embryonal | 3.59 (1.47-8.76) | 0.005 | 0.099 | **0.131** | 0.311 | 0.832 | 0.980 | 0.998 |
| High risk | 3.57 (1.28-9.90) | 0.014 | 0.133 | 0.246 | 0.495 | 0.915 | 0.991 | 0.999 |
| Trunk and limbs | 4.00(1.20-13.34) | 0.024 | 0.130 | 0.358 | 0.626 | 0.948 | 0.995 | 0.999 |
| Genitourinary system | 5.03(1.29-19.57) | 0.020 | 0.092 | 0.393 | 0.660 | 0.955 | 0.999 | 1.000 |
| Stage II | 9.95(2.73-36.23) | 0.001 | 0.007 | **0.165** | 0.372 | 0.867 | 0.985 | 0.998 |
| Stage IV | 4.65(1.19-18.22) | 0.027 | 0.113 | 0.421 | 0.686 | 0.960 | 0.996 | 1.000 |
| rs4946936 T > C | | | | | | | | |
| TC/CC vs. TT | 0.48 (0.25-0.90) | 0.022 | 0.450 | **0.129** | 0.334 | 0.847 | 0.982 | 0.998 |
| Age <10 years | 0.45 (0.24-0.86) | 0.016 | 0.375 | **0.111** | 0.269 | 0.802 | 0.976 | 0.998 |
| Male | 0.36 (0.17-0.78) | 0.010 | 0.202 | **0.125** | 0.295 | 0.822 | 0.979 | 0.998 |
| Medium risk | 0.33 (0.14-0.76) | 0.009 | 0.164 | **0.144** | 0.334 | 0.846 | 0.982 | 0.998 |
| Head and neck | 0.24(0.06-0.95) | 0.042 | 0.148 | 0.460 | 0.721 | 0.966 | 0.997 | 1.000 |
| Genitourinary system | 0.25(0.08-0.73) | 0.011 | 0.102 | 0.247 | 0.509 | 0.919 | 0.991 | 0.999 |
| Stage II | 0.16(0.05-0.50) | 0.002 | 0.025 | **0.163** | 0.393 | 0.877 | 0.986 | 0.999 |
| ^a^ Chi-square test was used to calculate the genotype frequency distributions.  ^b^ Statistical power was calculated using the number of observations in the subgroup and the OR and *P* values in this table.  The bold values were statistically significant results. | | | | | | | | |

| **Supplemental Table 4**. False-positive report probability analysis for the significant findings | | | | | | | | |
| --- | --- | --- | --- | --- | --- | --- | --- | --- |
| Genotype | Crude OR (95% CI) | *P* ^a^ | Statistical power^b^ | Prior probability | | | | |
|  |  |  |  | 0.25 | 0.1 | 0.01 | 0.001 | 0.0001 |
| rs17069665 A > G | | | | | | | | |
| GG vs. AA/AG | 2.96 (1.29-6.80) | 0.011 | 0.345 | **0.084** | 0.216 | 0.752 | 0.968 | 0.997 |
| Age <10 years | 2.98 (1.29-6.87) | 0.010 | 0.340 | **0.084** | 0.216 | 0.752 | 0.968 | 0.997 |
| Male | 3.33 (1.16-9. 57) | 0.026 | 0.297 | 0.205 | 0.436 | 0.895 | 0.988 | 0.999 |
| Embryonal | 3.59 (1.47-8.76) | 0.005 | 0.213 | **0.065** | **0.174** | 0.698 | 0.959 | 0.996 |
| High risk | 3.57 (1.28-9.90) | 0.014 | 0.247 | **0.150** | 0.345 | 0.853 | 0.983 | 0.998 |
| Trunk and limbs | 4.00(1.20-13.34) | 0.024 | 0.222 | 0.245 | 0.494 | 0.915 | 0.991 | 0.999 |
| Genitourinary system | 5.03(1.29-19.57) | 0.020 | 0.157 | 0.275 | 0.532 | 0.926 | 0.992 | 0.999 |
| Stage II | 9.95(2.73-36.23) | 0.001 | 0.018 | **0.076** | **0.197** | 0.730 | 0.965 | 0.996 |
| Stage IV | 4.65(1.19-18.22) | 0.028 | 0.187 | 0.306 | 0.569 | 0.936 | 0.993 | 0.999 |
| rs4946936 C > T | | | | | | | | |
| TT vs. CC/CT | 2.08 (1.10-3.95) | 0.025 | 0.713 | **0.096** | 0.241 | 0.778 | 0.972 | 0.997 |
| Age <10 years | 2.23 (1.16-4.26) | 0.015 | 0.635 | **0.067** | **0.177** | 0.703 | 0.960 | 0.996 |
| Male | 2.77 (1.28-5.98) | 0.009 | 0.397 | **0.067** | **0.177** | 0.702 | 0.960 | 0.996 |
| Medium risk | 3.57 (1.28-9.90) | 0.014 | 0.321 | **0.077** | 0.200 | 0.735 | 0.965 | 0.996 |
| Head and neck | 4.19(1.05-16.69) | 0.042 | 0.232 | 0.353 | 0.621 | 0.947 | 0.995 | 0.999 |
| Genitourinary system | 4.08(1.37-12.15) | 0.012 | 0.189 | **0.155** | 0.354 | 0.858 | 0.984 | 0.998 |
| Stage II | 6.43(2.00-20.69) | 0.002 | 0.057 | **0.087** | 0.223 | 0.759 | 0.970 | 0.997 |
| ^a^ Chi-square test was used to calculate the genotype frequency distributions.  ^b^ Statistical power was calculated using the number of observations in the subgroup and the OR and *P* values in this table. | | | | | | | | |

PROC SETINIT RELEASE=’9.4’;

SITEINFO NAME=‘SUNGKYUNKWAN UNIV-MIDTIER1 0401’

SITE=10502994 OSNAME=‘WX64_WKS’ RECREATE WARN=33 GRACE=0

BIRTHDAY=‘07APR2021’D EXPIRE=‘31MAR2022’D PASSWORD=194343661;

CPU MODEL=’ ’ MODNUM=’ ’ SERIAL=’ ’ NAME=CPU000;

EXPIRE ‘PRODNUM000’ ‘PRODNUM001’ ‘PRODNUM002’ ‘PRODNUM003’

‘PRODNUM004’ ‘PRODNUM005’ ‘PRODNUM006’ ‘PRODNUM007’

‘PRODNUM008’ ‘PRODNUM010’ ‘PRODNUM013’ ‘PRODNUM015’

‘PRODNUM025’ ‘PRODNUM035’ ‘PRODNUM050’ ‘PRODNUM070’

‘PRODNUM075’ ‘PRODNUM094’ ‘PRODNUM095’ ‘PRODNUM119’

‘PRODNUM123’ ‘PRODNUM164’ ‘PRODNUM165’ ‘PRODNUM166’

‘PRODNUM167’ ‘PRODNUM192’ ‘PRODNUM194’ ‘PRODNUM204’

‘PRODNUM208’ ‘PRODNUM209’ ‘PRODNUM215’ ‘PRODNUM216’

‘PRODNUM219’ ‘PRODNUM222’ ‘PRODNUM225’ ‘PRODNUM448’

‘PRODNUM535’ ‘PRODNUM538’ ‘PRODNUM550’ ‘PRODNUM555’

‘PRODNUM557’ ‘PRODNUM560’ ‘PRODNUM561’ ‘PRODNUM563’

‘PRODNUM564’ ‘PRODNUM565’ ‘PRODNUM566’ ‘PRODNUM567’

‘PRODNUM568’ ‘PRODNUM677’ ‘PRODNUM678’ ‘PRODNUM884’

‘PRODNUM964’ ‘PRODNUM1304’ ‘PRODNUM1312’ '31MAR2022’D

/ CPU=CPU000;

SAVE; RUN;

Ref: TCCTGTCTTTGAGGTCTTAACGTGGTTTC**A**TGTGCCTTTTGATTTGTTTTTACATGTGG

Alt: TCCTGTCTTTGAGGTCTTAACGTGGTTTC**G**TGTGCCTTTTGATTTGTTTTTACATGTGG
