# Supplementary figures and images for: FOXO3 polymorphisms influence the risk and prognosis of rhabdomyosarcoma in children
Source: Front Oncol. 2024 Apr 24;14:1387735. doi: 10.3389/fonc.2024.1387735 (PMC11076676; doi:10.3389/fonc.2024.1387735)

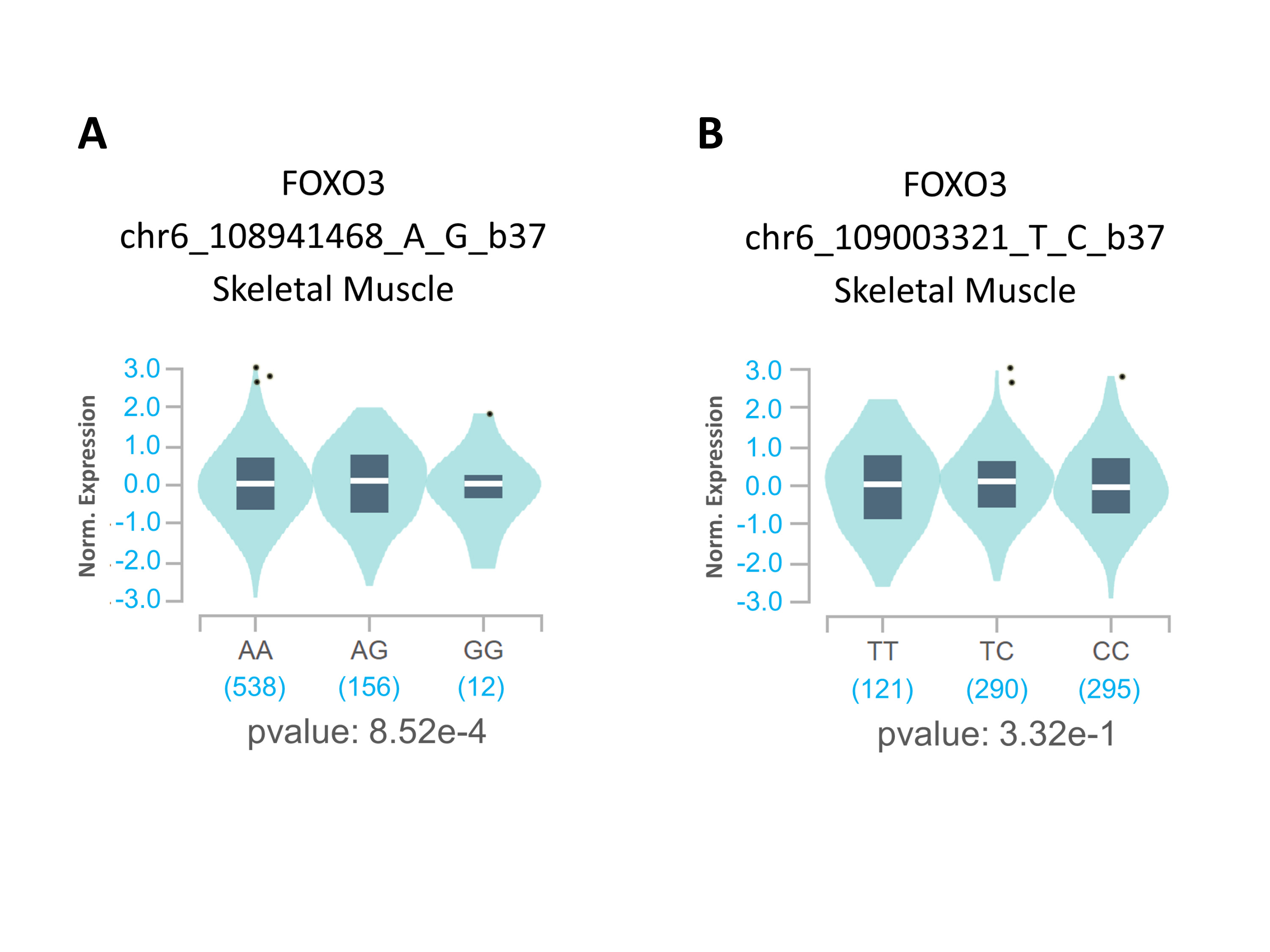

Supplement: Supplementary Figure 1 — Functional relevance of rs3738067 A>G (A) and rs4946936 T > C (B) to FOXO3 expression in GTEx database. [file Image_1.jpeg]

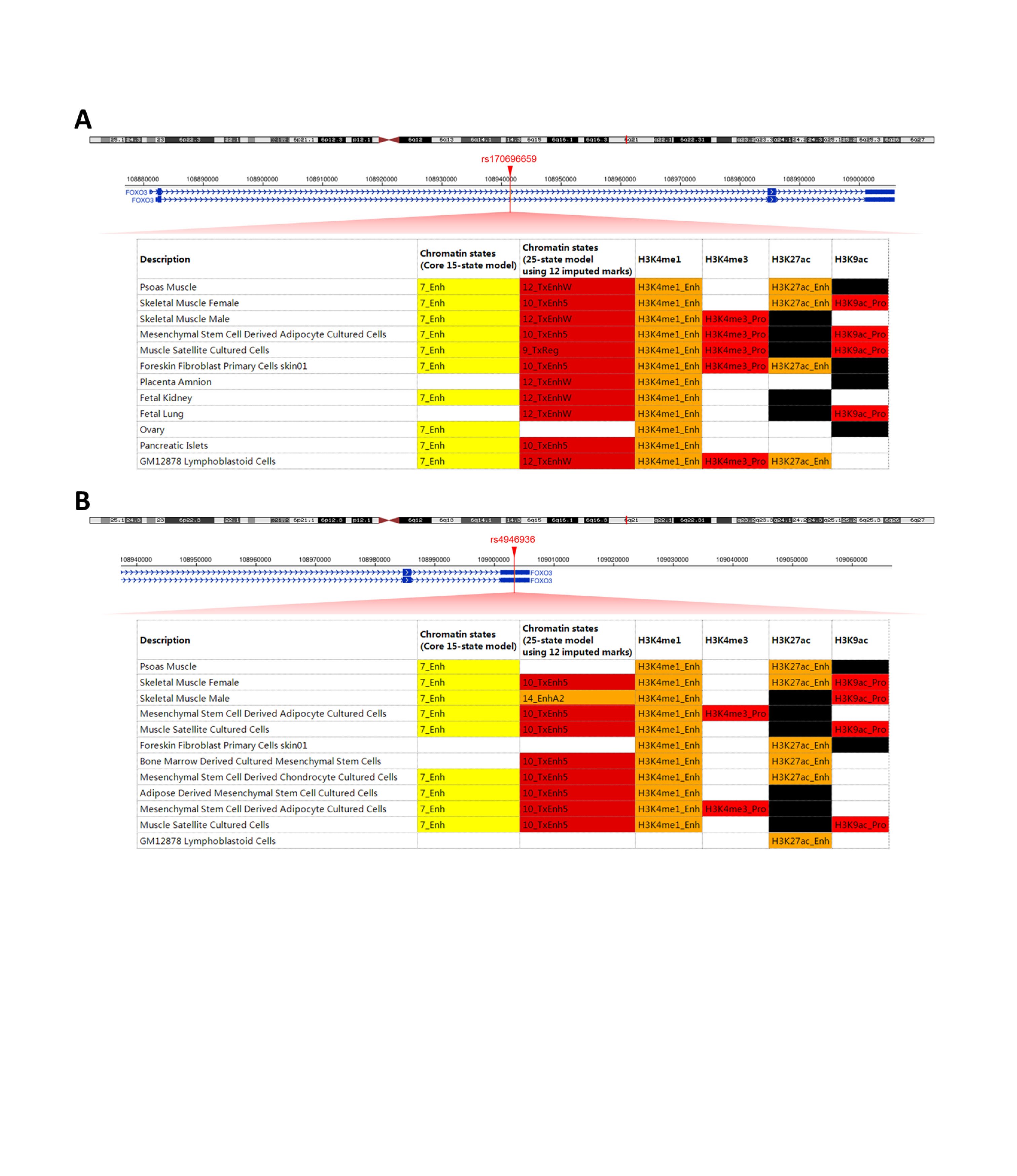

Supplement: Supplementary Figure 2 — Schematic of rs17069665 (A) and rs4946936 (B) regions with histone and DHS mark annotations in different tissue types in the Roadmap epigenomics data. [file Image_2.jpeg]
